# How transcriptional networks control *Verticillium* plants infection

**DOI:** 10.1371/journal.ppat.1013673

**Published:** 2025-11-10

**Authors:** Ying-Yu Chen, Rebekka Harting, Gerhard H. Braus

**Affiliations:** Department of Molecular Microbiology and Genetics, Institute of Microbiology and Genetics and Göttingen Center for Molecular Biosciences (GZMB), University of Göttingen, Göttingen, Germany; University of Tübingen: Eberhard Karls Universitat Tubingen, GERMANY

## What is the significance of plant diseases caused by *Verticillium* spp.?

Phytopathogenic fungi of the ascomycete genus *Verticillium* are able to induce widespread and economically significant plant diseases, which result in agricultural loss [1,2]. The host range of the amphidiploid *V. longisporum* is relatively narrow and mainly includes Brassicaceae plants [1,3]. It induces the so-called Verticillium stem striping, for example, in rapeseed plants. In contrast, the haploid relative *V. dahliae* is able to infect many different hosts, including tomato, strawberry, sunflower plants, and olive trees, resulting in Verticillium wilt disease [1,2]. The *ex planta* life of *Verticillium* is predominantly in the form of dormant resting structures, the microsclerotia, followed by the relatively short germination and infection period. Infection starts with fungal hyphae getting in contact with the host plant through roots. When *Verticillium* reaches the xylem, it produces asexual conidiospores, which get distributed throughout the plant. Blockage of the plant vasculature transport leads to the typical symptoms. Germination of conidiospores allows the fungus to systemically colonize its host. *V. dahliae* and *V. longisporum* both form microsclerotia when the infected plants are dying. Microsclerotia are released into the soil and are protected from environmental stresses by a melanin layer. This allows the fungus to bridge the time until a new host plant can be infected [1,2]. Conventional fungicides were shown to be ineffective once the fungus is in the plant vasculature or in dormancy, and the most effective way for the control of Verticillium wilt was by fumigating the soil with methyl bromide, a method that is no longer used due to environmental concerns [2].

## How do *Verticillium* spp. enter the plant hosts?

Adhesion of *Verticillium* hyphae to plant root surfaces represents the critical transition from its short, saprophytic phase in the soil to host colonization. As a soil-borne pathogen, *V. dahliae* must maintain stable contact with root surfaces despite environmental disturbances such as water flow or soil movement. This adhesive interaction likely facilitates successful root penetration and the establishment of infection. The ability to adhere is also essential for single-celled or filamentous fungi to initiate a transition to a new lifestyle [4,5]. In *Saccharomyces cerevisiae*, cell-cell adhesion contributes to the switch from single cellular growth to multicellular flocs (flocculation), and cell-abiotic surface adhesion can be induced by various starvation conditions and leads to multicellular filaments or biofilms [5,6]. To elucidate the molecular regulation of the sequential events that enable *Verticillium* spp. to adhere and colonize its host, the non-adhesive *S. cerevisiae* ∆*FLO8* strain was used to perform a forward genetic screen with *V. longisporum* cDNA [7]. *Verticillium* transcriptional activator of adhesion 1-6 (Vta1-6) and *Verticillium dahliae* Som1, the direct counterpart of the yeast transcription factor Flo8, were shown to be able to activate the yeast adhesin encoding genes. Som1 was also required for *Verticillium* adhesion to abiotic surfaces [7,8]. Som1, Vta3, and Vta2 reflect the control of sequential steps of *Verticillium*’s host infection with the transition from the *ex planta* to *in planta* life. Som1 and Vta3 were shown to be required for earlier steps in plant root colonization [8]. Deletion of *SOM1* resulted in a strain unable to adhere to the root surface and propagate there. Strains without *VTA3* grew on root surfaces but were impaired in early propagation [8]. Vta2 was dispensable for early propagation on root surface but needed for systemic colonization of roots [7]. The chronological order of these involvements is reflected in the genetic regulatory network. Som1 controls the expression of Vta3, and both Som1 and Vta3 regulate the later *VTA2* subnetwork [8]. The *SOM1*, *VTA3*, or *VTA2* deletion strains were unable to induce plant disease symptoms. Stress conditions during plant infection can cause phytopathogenic fungi to produce an increased number of unfolded or misfolded proteins. The unconventional splicing of *HAC1* mRNA triggers unfolded protein response, and the transcription factor Hac1 activates a subset of genes in response. In *V. dahliae*, the deletion of *HAC1* resulted in less hyphae on plant root surfaces [9]. Since both orthologs of Som1 and Hac1 regulate the expression of an adhesin-encoding gene in yeast, the Som1 regulatory network might be interlinked to unfolded protein responses [9,10].

## How are *Verticillium* spp. developmental processes coordinated *in planta*?

**Fig 1:**
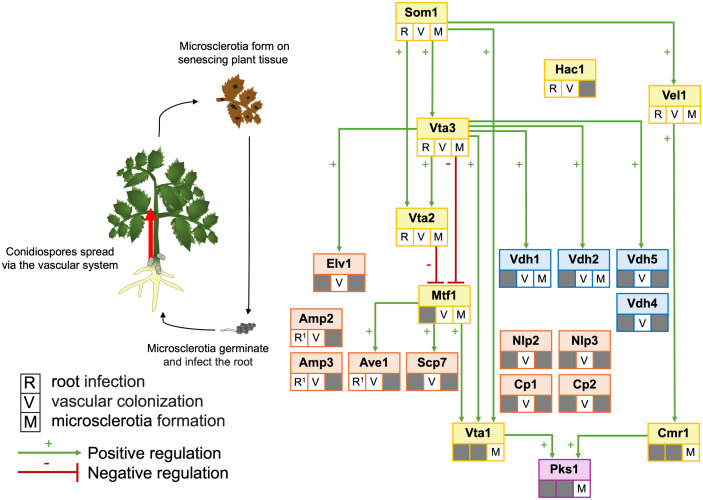
The genetic regulatory network of *Verticillium dahliae.* The proteins are depicted according to the known involvement in root infection (R), vascular colonization (V), and microsclerotia formation (M). R^1^ indicates effectors that indirectly contribute to root infection by inhibiting soil microbiota. Green arrows indicate a positive regulatory relationship in transcript levels, and red bars indicate negative regulatory relationships. Yellow, orange, blue, and purple boxes indicate transcription factors, effectors, hydrophobins, and biosynthetic enzymes, respectively.

Developmental processes beyond fungal entry into plants are also controlled by the Som1-Vta regulatory network (Fig 1). Vta2 is a positive regulator of vegetative growth and conidiospore production. Vta2 is also required for the correct timing of microsclerotia formation. As in its absence, resting structure production was observed earlier compared to the control strains [7]. The Master transcription factor 1 (Mtf1) is repressed by Vta2 and Vta3. It is shown to be involved in virulence towards plant hosts and microsclerotia formation by promoting the expression of *VTA1* [11]. In contrast to Vta3 and Vta2, Vta1 is not involved in the plant infection process [12]. Neither plant root colonization nor induction of plant disease symptoms was altered when Vta1 was absent from the cells. However, albino microsclerotia lacking the protective melanin were produced when *VTA1* was deleted. The transcription factor is located in the melanin biosynthesis cluster, and it was shown to regulate melanin biosynthesis genes such as the polyketide synthase encoding *PKS1* [13]. Reduced conidiation and microsclerotia formation were also observed in the ∆*HAC1* mutant strain, indicating that unfolded protein response is also important for the fungus to mitigate stress conditions during *in planta* growth [9]. The velvet regulators are conserved in the fungal kingdom, and coordinate development with secondary metabolism [14]. Som1 positively regulates Vel1, which is involved in plant root penetration, microsclerotia formation, conidiation, and secondary metabolism [8,14]. Contrary to the Mtf1 regulation, Vel1 governs microsclerotia formation through *CMR1*, a transcription factor encoding gene that is localized in the melanin biosynthesis gene cluster as *VTA1* [12]. Vta1 and Cmr1 operate independently, but both positively regulate melanin biosynthesis through *PKS1* [12,13].

## What is the contribution of secreted proteins to *Verticillium* development and infection?

Hydrophobins are small proteins that are often present on the surface of fungal tissues to reduce surface tension between hyphae and the environment [15]. The *in planta* growth of Verticillia involves surviving and rapidly spreading in the nutrient-poor plant xylem sap. To date, four hydrophobin-encoding genes, *VDH1-2* and *VDH4-5,* have been studied in *V. dahliae*, all of which had higher transcript levels in tomato xylem sap compared to pectin-rich medium [16]. RNA-seq experiments revealed that Vta3 positively regulates the expression of hydrophobin genes *VDH1*, *VDH2* (also published as *VdHP1*), and *VDH5* [11,16]. *VDH4* and *VDH5* contribute to pathogenicity, which aligns with the higher transcript levels in the xylem sap [16]. *VDH1* and *VDH2* were reported to contribute to microsclerotia development in certain *V. dahliae* isolates, whereas *VDH2* was also shown to impact virulence towards cotton plants [17,18].

Effectors are another type of proteins secreted by phytopathogens to facilitate pathogenicity. Several effector-encoding genes contribute to pathogenicity of *Verticillium* spp. and are coordinated by the Som1-Vta-Mtf1 network, such as the Vta3-regulated *ELV1*, and Mtf1-regulated *AVE1* and *SCP7* [11]. Common roles of effectors include the manipulation of host microbiota, the disruption of host cell integrity or host physiological processes, and the suppression of host immune response. *Verticillium* hyphae grow towards the plant host upon germination from dormancy, and the rhizosphere microbiota act as first barrier to resist fungal pathogens from infecting the plant. The composition of the soil microbiome is shaped by the plant to suppress pathogens, as well as by phytopathogens to inhibit antagonists [19]. Effectors secreted by *V. dahliae* to target bacterial competitors include Ave1, Amp2, and Amp3 [19]. Upon entry into the plant host, the fungus encounters barriers of plant cellular structures and the plant immune response. Carbohydrate active enzymes (CAZymes) and cytotoxic effectors were enriched in the exoproteome of *V. longisporum* cultured in rapeseed (*Brassica napus*) xylem sap, and CAZymes were also enriched in the exoproteome of *V. dahliae* cultured in cotton-containing minimal medium [20,21]. CAZymes are secreted to degrade plant cell wall components such as cellulose and pectin, whereas other effectors, such as Nlp2 and Nlp3, cause necrosis by triggering plant defense responses, and Cp1, and Cp2 degrade host defense proteins such as chitinases from targeting the fungal cell wall [21–23].

## Conclusion

The basic adhesion system of *S. cerevisiae,* as dimorphic organism, can be used to gain insights into processes of more complex filamentous fungi, such as host infection of *Verticillium* spp. The regulators of adhesion have evolved more complex roles in filamentous fungi and are able to control complex developmental steps with secondary metabolism and virulence processes. Current control methods of Verticillium wilt are limited in effectiveness due to resistant microsclerotia and the rapid spread of conidiospores in the plant vasculature system [2]. An improved understanding of regulatory networks that govern key developmental process may provide new targets for future plant protection measures.
